# Supplementary material for: Preparation and Characterization of a Nano-Inclusion Complex of Quercetin with β-Cyclodextrin and Its Potential Activity on Cancer Cells
Source: Micromachines (Basel). 2023 Jun 30;14(7):1352. doi: 10.3390/mi14071352 (PMC10386393; doi:10.3390/mi14071352)
Supplement: Supplementary file 1 [file micromachines-14-01352-s001.zip › micromachines-2441067-supplementary.pdf]

Preparation and Characterization of a Nano-Inclusion Complex of Quercetin with β-Cyclodextrin and Its Potential Activity on Cancer Cells

Rajaram Rajamohan [1,*], Sekar Ashokkumar [2], Kuppusamy Murugavel [3] and Yong Rok Lee [1,*]

[1] Organic Materials Synthesis Laboratory, School of Chemical Engineering, Yeungnam University, Gyeongsan 38541, Republic of Korea
[2] Plasma Bioscience Research Center, Kwangwoon University, Seoul 01897, Republic of Korea; kumarebt@gmail.com
[3] PG & Research Department of Chemistry, Government Arts College, Chidambaram 608 102, Tamil Nadu, India; ksmvel@gmail.com
* Correspondence: rajmohanau@yu.ac.kr (R.R.); yrlee@yu.ac.kr (Y.R.L.)

Lungs fibroblasts MRC-5 and triple-negative breast cancer MB 231 cell culture

The MRC-5 and MDA-MB-231 cells are cultured in a T-75 flask containing Dulbecco's Modified Eagle Medium (DMEM) supplemented with 10% fetal bovine serum and 1% antibiotic (streptomycin & penicillin). The cells inoculated plates are incubated at 37 °C in 5% CO_2 for up to 2 days, and the cells reached ~80% confluence to start the cell viability experiment.

Cytotoxicity (*in-vitro* cell viability)

The *in-vitro* cell viability of the QRC and NICs is investigated by Alamar blue assay. Briefly, MRC-5 and MDA-MB-231 cells are seeded into 96 well plates at a density of 2×10^4 cells/well when cells reached 80% confluence in 1 day. Then, the growth medium (DMEM) was removed and one time washed with PBS. After PBS washing, 200 µl of fresh medium (DMEM) containing different concentrations of derivatives (0.2, 2, 20, 200, and 2000 µg/ml) is added into each well and incubated for up to 3 days. Based on diverse instance periods, the cells are washed with PBS and further added 100 µl of 10% of Alamar blue, incubated at 37 °C with 5% CO_2 for up to 4 hours. After incubation plate was read at excitation and emission wavelength of 540 and 600 nm respectively using a microplate reader Synergy HT spectrophotometer (BioTek, Winooski, VT, USA).

Apoptosis study

MRC-5 and MDA-MB-231 cells are seeded into 6 well plates at a density of 2×10^5 cells/well and the cells reached 80 to 90% confluence. The culture medium is removed and one time washed with PBS. After washing fresh medium containing 1 mg/ml of QRC, NICs are incubated for 4 hrs, in blank without derivatives. Floating and adherent cells are collected and centrifuged at 1500 rpm for 5 min, and the pellets are washed twice with cold PBS solution and resuspended. The cells are added 100 µl of 1X binding buffer, 5.0 µl of Annexin V-FITC, and propidium iodide (PI) respectively, and incubated for 15 minutes at room temperature. In the end, cell apoptosis is measured with flow cytometry (BD FACSVerse, BD Biosciences, NJ, USA).

Instruments Used

Various techniques are employed to analyze the NICs, including field-emission scanning electron microscopy (FE-SEM), high-resolution transmission electron microscopy (HR-TEM), dynamic light scattering (DLS), powder X-ray diffraction (XRD), differential scanning calorimetry (DSC), Fourier transform infrared spectroscopy (FT-IR), Raman spectroscopy, proton (^{1}H) and carbon-13 (^{13}C) nuclear magnetic resonance (NMR) spectroscopy, and rotating frame overhauser effect spectroscopy (ROESY).

FE-SEM analysis is conducted at an accelerating voltage of 10.0 kV using the Hitachi S-4800 instrument equipped with energy-dispersive X-ray spectroscopy (EDX). HR-TEM images are acquired with an FEI-Tecnai TF-20 transmission electron microscope operating at an accelerating voltage of 120.0 kV. For DLS measurements, a Zetasizer Nano S system by Malvern Instruments, UK, is employed to analyze a sample of colloidal gold. The experiments are carried out at a temperature of 25 °C using a wavelength of 633 nm and an avalanche photodiode (APD) detector. The scattered light is detected at an angle of 173 degrees. Powder XRD measurements are performed using a PANalytical X'Pert3 MRD diffractometer at 40.0 kV and 30 mA. The X-ray radiation is monochromatized with Cu K radiation (λ = 1.54 Å). The measurement range covers 2θ angles from 10° to 80°, with a scan rate of 5° min^{-1} and a wavelength of 1.5405 Å. Thermal analysis of the samples is conducted using TA Instruments, and the Universal V4.5A Program is utilized for analyzing the thermal curves. Each sample weighs 3.4 mg, and the temperature range for the analysis is 40.0 to 250.0 °C, with a temperature ramp rate of 10.0 °C/minute. FT-IR spectra are recorded on a Perkin Elmer Spectrum Two spectrometer in transmittance mode, covering a range of 400.0 to 4000.0 cm^{-1}. A resolution of 8 cm^{-1} is used, and each spectrum is an average of 8 scans. Raman spectral measurements are acquired using the XploRA Micro-Raman spectrophotometer by Horiba. The measurements are taken in the range of 100.0 to 1500.0 cm^{-1}. The ^{1}H, ^{13}C NMR, and ROESY analyses of the NICs sample are performed using a Bruker

600 MHz NMR spectrometer. All of these instruments and services are availed at the core research support center (CRSC) for natural products and medical materials at Yeungnam University, South Korea.

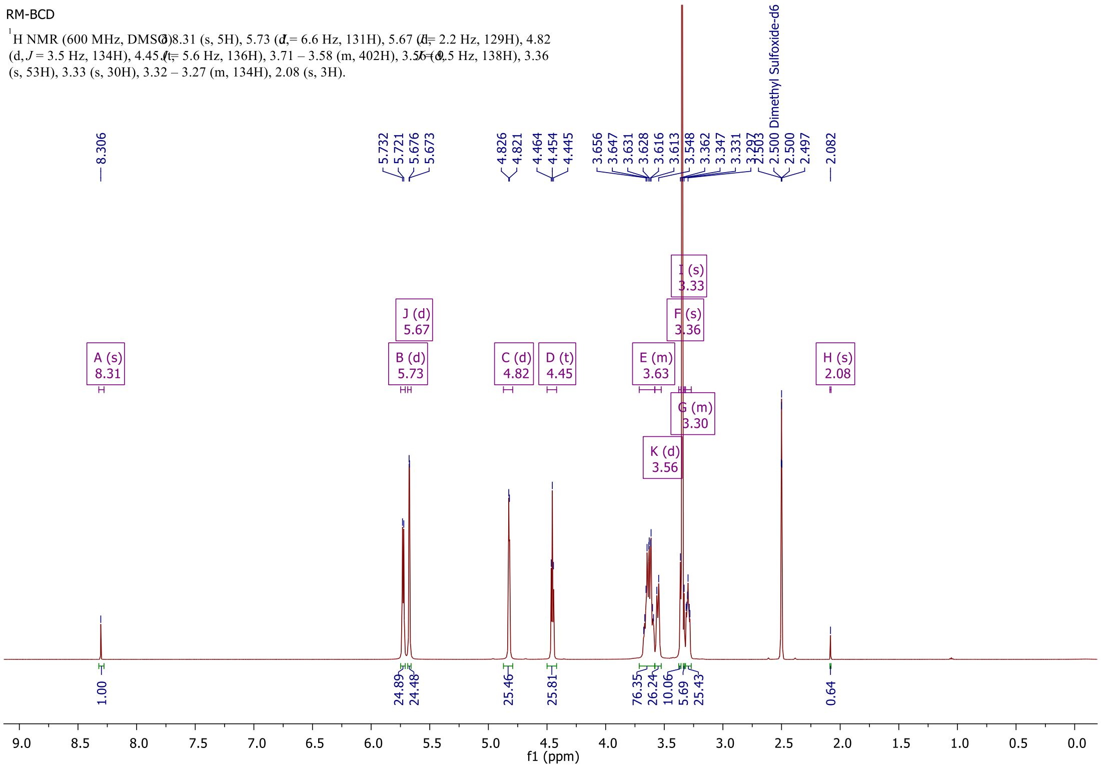

Figure S1. ^{1}H NMR spectra of β-CD.

^{13}C NMR spectral analysis of QRC

The carbon-13 spectrum of the quercetin shows, signals in the region of 90-180 ppm. The individual assignments of the signals were done by considering the substituent effect of the substituent and the position and intensity of the carbon signals. Among the ^{13}C signals, the signal at 175. 8 ppm is assigned to the carbonyl carbon (C-1) of the benzopyran ring. The ^{13}C signals at 163.9 and 160.7 ppm are assigned to the ipso carbons of the benzoxopyran ring. Hence, these may be due to the C-6 and C-

8 carbons. The ^{13}C signals at 147.7, 146.8, and 121.9 ppm are assigned to the ipso carbons of the phenyl ring attached to the benzoxopyran ring. These signals may be due to C-3' C-4' and C-1'. The ^{13}C signals at 156.1 and 135.7 ppm are assigned to C-3 and C-2 carbons respectively. More up-field ^{13}C signals observed at 93.3 and 98.2 ppm are assigned to C-7 and C-5, respectively. The ^{13}C signals at 115.0, 115.6, and 119.9 ppm may be due to the signals of the phenyl ring attached to benzoxopyran and these are assigned to C-2', C-5', and C-6'. The other two ^{13}C signals at 145.0 and 103.0 ppm are conveniently assigned to C-10 and C-9, respectively. The C-13 chemical shifts are shown in **Table S4.**

^{13}C NMR spectral analysis of QRC:β-CD NICs

Similar to Proton chemical shifts, the ^{13}C chemical shifts are also assigned by taking into consideration of shielding, and substituent effect and by comparing the QRC and β-CD values. The carbon -13 chemical shifts are not affected by complex formation, hence there is no change in the chemical shifts. The ipso carbons (whose peak height is less) of the quercetin molecules are observed at 163.9, 160.7, 121.9, 146.8, and 147.7 ppm, and these are assigned to C-6, C-8, C-1', C-3' and C-4', respectively. ^{13}C signals observed at 175.8, 135.7, and 156.1 ppm are assigned to carbonyl carbon C-1, C-2, and C-3, respectively. Other signals of quercetin molecules were observed in the range of 93.3 - 119.9 ppm. In the ^{13}C spectrum, the signal observed at 101.9 ppm is assigned to the methine carbon of the β-CD having an equatorial hydrogen atom. The other methine carbons of the β-CD's were obtained at 81.5, 78.9, 73.0, and 72.4 ppm. carbon-13 signal observed at 59.9 ppm is conveniently assigned to methylene carbon. The carbon-13 chemical shifts are collected in **Table S4**.

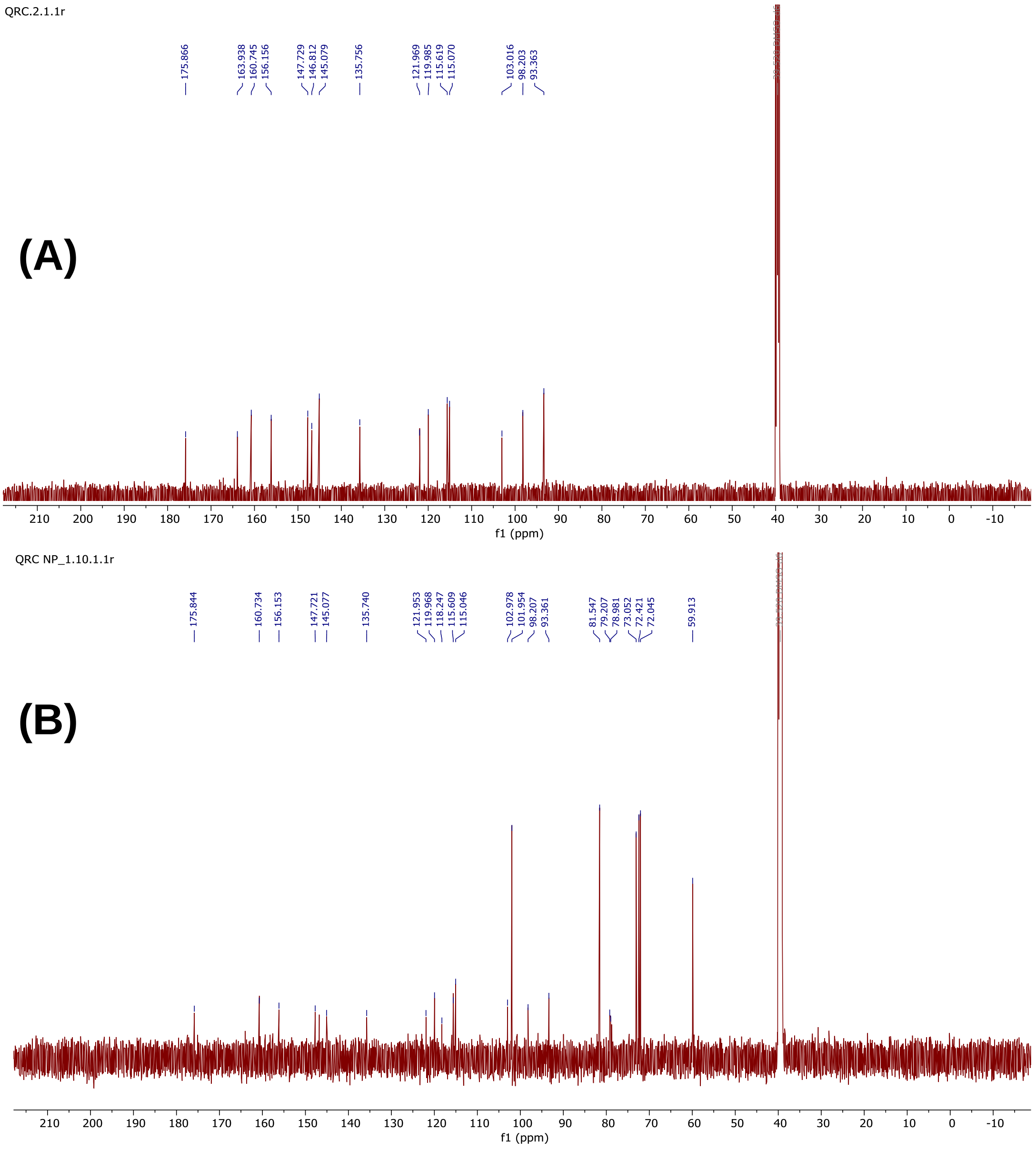

Figure S2. ^{13}C NMR chemical shifts of QRC **(A)**, and NICs **(B).**

Table S1. DLS data of QRC:β-CD NICs

Sample	Particle Size, nm	% Intensity	Standard Deviation	PDI
NICs	206.7 ± 4.15	100	28.13	0.833

Table S2. FT-IR spectra of QRC, and QRC:β-CD NICs

S. No	Possible Vibrations	QRC (cm^{-1})	β-CD [1,2] (cm^{-1})	NICs (cm^{-1})
1.	O-H st	3410, 3291	3300	3306
2.	C-H (Asymmetric)	-	2930	2922
3.	C-C	-	1650	1656
4.	C-H bending	-	1480-1180	
5.	C-O-C, C-O (Symmetric of glycosidic	-	1200-1000	1154, 1079, 1025
6.	O-H bending	1369	-	1370
7.	C=O	1652	-	1656
8.	C-O str	1256	-	1260
9.	C-O (phenolic)	1196	-	1199
10.	C-CO-C st	1156	-	1154

Table S3. ^{1}H NMR chemical shifts of QRC, β-CD, and NICs

Positions	QRC		β-CD		NICs	
	**Chemical shifts (ppm)**	**Multiplicities**	**Chemical shifts (ppm)**	**Multiplicities**	**Chemical shifts (ppm)**	**Multiplicities**
OH protons at equatorial positions	-	-	4.82, 5.67	Doublet of doublet	4.89, and 5.75 (merged)	Doublet (3 Hz), doublet (1.8 Hz)
Methine proton axial (CH_2OH attached proton)	-	-	4.45	Triplet	4.51	Triplet (11.4 Hz)
CH and CH_2 protons	-	-	3.30, 3.56 and 3.63	Multiplet.	3.33, 3.62, and 3.70	Multiplet.
OH of CH_2OH	-	-	3.33	multiplet	3.33	multiplet
Equatorial hydrogen	-	-	5.73	Doublet	5.75 (merged)	Doublet (6.6 Hz)
OH at C-2	12.48	singlet	-	-	12.54	singlet
Hydroxy protons at C-6 and C-8	10.80, 9.60	Broad singlet	-	-	10.66, 9.42 (merged)	Broad singlet

H-5	6.18	Doublet (1.8 Hz)	-	-	6.23	Doublet (1.8 Hz)
H-7	6.40	Doublet (1.8 Hz)	-	-	6.45	Doublet (1.8 Hz)
Hydroxy protons at C-3' and 4'	9.35	Broad singlet	-	-	9.42	Broad singlet
H-2'	7.67	Doublet (2.4 Hz)	-	-	7.73	Doublet (2.4 Hz)
H-5'	7.53	Doublet of doublet (8.4 & 2.4 Hz)	-	-	7.60	Doublet of doublet (8.4 & 2.4 Hz)
H-6'	6.88	Doublet (8.4 Hz)	-	-	6.93	Doublet (8.4 Hz)

Table S4. ^{13}C NMR chemical shifts of QRC, and NICs

QRC		NICs
**Positions**	**Chemical shifts (ppm)**	**Chemical shifts (ppm)**
C-1	175.8	175.8
C-2	135.7	135.7
C-3	156.1	156.1
C-5	98.2	98.2
C-6	163.9	163.9
C-7	93.3	93.3
C-8	160.7	160.7
C-9	145.0	145.0
C-10	103.0	103.0
C-1'	121.9	121.9
C-2'	115.0	115.0
C-3'	146.8	146.8
C-4'	147.7	147.7
C-5'	115.6	115.6
C-6'	119.9	119.9
Methine carbons (bearing OH at equatorial)	-	72.4, 78.9, & 81.5
Methine carbon (CH_2OH attached)	-	73.0
Methylene carbon	-	59.9
Methine carbon (bearing equatorial hydrogen)	-	101.9

Table S5. The cytotoxic ability of QRC, and QRC:β-CD NICs on cancer cell lines.

Name of the sample	Concentration (μg/ml)	MDA MB 231				MCF-7			
		Day 1		Day 3		Day 1		Day 3	
		% of survival	SD	% of survival	SD	% of survival	SD	% of survival	SD
	**Control**	100	0	100	0	100.00	0.00	100.00	0.00
**QRC**	**0.2**	93.5	0.20	90.5	0.32	98.39	0.27	97.08	0.28
	**2**	88.0	1.22	81.9	1.62	97.96	0.01	97.07	0.13
	**20**	84.7	0.49	58	0.81	97.12	0.55	96.88	0.15
	**200**	80.1	0.81	51.7	0.65	96.49	0.41	80.93	0.78
	**2000**	78.4	1.01	27.8	1.62	93.68	0.02	84.03	0.43
**NICs**	**Control**	100.0	0.00	100	0.00	100.00	0.00	100.00	0.00
	**0.2**	98.1	0.18	98.4	0.08	99.00	0.69	98.28	0.40
	**2**	97.2	1.01	97.5	0.62	98.92	0.14	98.03	0.28
	**20**	94.4	3.63	92.5	1.47	98.26	0.76	97.86	0.13
	**200**	88.6	3.97	86.1	0.71	97.83	0.43	96.81	0.61
	**2000**	64.6	15.31	65.9	14.58	97.09	0.35	95.73	0.63

*SD : Standard Deviation
